# Analysis of Intracellular Communication Reveals Consistent Gene Changes Associated with Early-Stage Acne Skin

**DOI:** 10.21203/rs.3.rs-4402048/v1

**Published:** 2024-05-29

**Authors:** Min Deng, Woodvine O. Odhiambo, Min Qin, Thao Tam To, Gregory M. Brewer, Alexander R. Kheshvadjian, Carol Cheng, George W. Agak

**Affiliations:** University of California (UCLA); University of California (UCLA); University of California (UCLA); University of California (UCLA); University of California (UCLA); University of California (UCLA); University of California (UCLA); University of California (UCLA)

**Keywords:** Cell-cell communication, acne vulgaris, signal distribution, Cutibacterium acnes, single cell and spatial transcriptomic sequencing, inflammation, TREM2 macrophages, GRN, hyperkeratinization, IL-13RA1

## Abstract

A comprehensive understanding of the intricate cellular and molecular changes governing the complex interactions between cells within acne lesions is currently lacking. Herein, we analyzed early papules from six subjects with active acne vulgaris, utilizing single-cell and high-resolution spatial RNA sequencing. We observed significant changes in signaling pathways across seven different cell types when comparing lesional skin samples (LSS) to healthy skin samples (HSS). Using CellChat, we constructed an atlas of signaling pathways for the HSS, identifying key signal distributions and cell-specific genes within individual clusters. Further, our comparative analysis revealed changes in 49 signaling pathways across all cell clusters in the LSS– 4 exhibited decreased activity, whereas 45 were upregulated, suggesting that acne significantly alters cellular dynamics. We identified ten molecules, including GRN, IL-13RA1 and SDC1 that were consistently altered in all donors. Subsequently, we focused on the function of GRN and IL-13RA1 in TREM2 macrophages and keratinocytes as these cells participate in inflammation and hyperkeratinization in the early stages of acne development. We evaluated their function in TREM2 macrophages and the HaCaT cell line. We found that GRN increased the expression of proinflammatory cytokines and chemokines, including IL-18, CCL5, and CXCL2 in TREM2 macrophages. Additionally, the activation of IL-13RA1 by IL-13 in HaCaT cells promoted the dysregulation of genes associated with hyperkeratinization, including KRT17, KRT16, and FLG. These findings suggest that modulating the GRN-SORT1 and IL-13-IL-13RA1 signaling pathways could be a promising approach for developing new acne treatments.

## Introduction

Acne vulgaris, the most common dermatological condition worldwide, presents as a chronic inflammatory and recurrent disease marked by a spectrum of lesions, including non-inflamed (both open and closed comedones) and inflamed (macules, papules, pustules, and nodules). Approximately 95% of boys and 85% of girls experience acne during adolescence, with nearly half of them experiencing it to adulthood^1^. The scarring and post-inflammatory hyperpigmentation caused by acne can severely affect an individual’s quality of life, highlighting the importance of early and effective therapy. The development of acne is driven by four key processes within the pilosebaceous unit (PSU): inflammation, epithelial hyperkeratinization, hyperseborrhea accompanied by proinflammatory lipids, and colonization by *Cutibacterium acnes* (*C. acnes*) bacteria^1^. Despite significant progress in elucidating the pathophysiology and treatment mechanisms of acne, it is important to identify the shared dysregulated signaling pathways in individuals with acne. Targeting these shared signaling pathways can significantly improve the effectiveness of current acne treatments in affected patients.

Skin homeostasis relies on a sophisticated network of resident cells, each performing unique biological functions and engaging in complex signaling pathways mediated by intricate ligand-receptor interactions^2–5^. The epidermis hosts various cells such as keratinocytes, melanocytes, Langerhans cells and merkel cells. Among them, basal keratinocytes serve as epithelial stem cells, crucial for proliferation and differentiation, ensuring the daily renewal of the epidermis. In the dermis and hypodermis, a diverse array of cells including fibroblasts, immune cells, endothelial cells, nerves, and adipocytes form a harmonized network. Dysregulated signaling among these cells has been implicated in various skin disorders such as vitiligo, impaired wound healing, aging, psoriasis, and dermatitis^2–5^. In acne lesions, excessive squalene production by keratinocytes and sebocytes triggers TREM2 macrophage differentiation, enhancing immune cell migration and fueling the inflammatory cascade^6^. Moreover, sebocytes adjacent to the PSU in acne lesions release CXCL8, attracting neutrophils, monocytes, and T cells, in addition to secreting IL-6, TGF-β, and IL-1β, which drive the differentiation of T helper 17 cells (Th17 cells)^7^. Our previous studies demonstrated that *C. acnes* ribotypes differentially regulate the fate of Th17 responses in the skin^8,9^. However, the comprehensive and conserved changes in intercellular communication within acne-affected skin are yet to be thoroughly investigated.

In this study, we examined global changes in intercellular communication by analyzing single cell RNA sequencing (scRNA-seq) and spatial transcriptomic data from six patients with papular acne. Our findings reveal that acne triggers significant alterations in 49 signaling pathways across all skin cell clusters compared to nonlesional areas. We also identified 10 genes related to these signaling pathways that were consistently dysregulated in all donors. Our focus was primarily drawn to genes that were enriched and upregulated in keratinocytes and immune cells, particularly myeloid cells and lymphocytes, as they play key roles in inflammation and hyperkeratinization during the onset of acne. Among these genes, we observed significant upregulation of GRN and IL-13RA1 within TREM2-expressing macrophages and basal keratinocytes, respectively. Furthermore, we found that GRN and its receptor SORT1 were upregulated in IL-4-induced TREM2 macrophages, and treatment with GRN led to increased expression of proinflammatory cytokines and chemokines from these cells. Concurrently, treating human HaCaT cells with IL-13 to activate IL-13RA1 signaling resulted in dysregulation of *KRT16, KRT17*, and *FLG* expression, which are associated with hyperproliferation-associated phenotypes in acne. Our findings suggest that GRN and IL-13RA1 are key players in the inflammation and hyperkeratinization process during acne development, highlighting their potential as novel therapeutic targets for acne treatment.

## Results

### Overall cell-cell communications and signal distributions in normal skin

**Figure 1:**
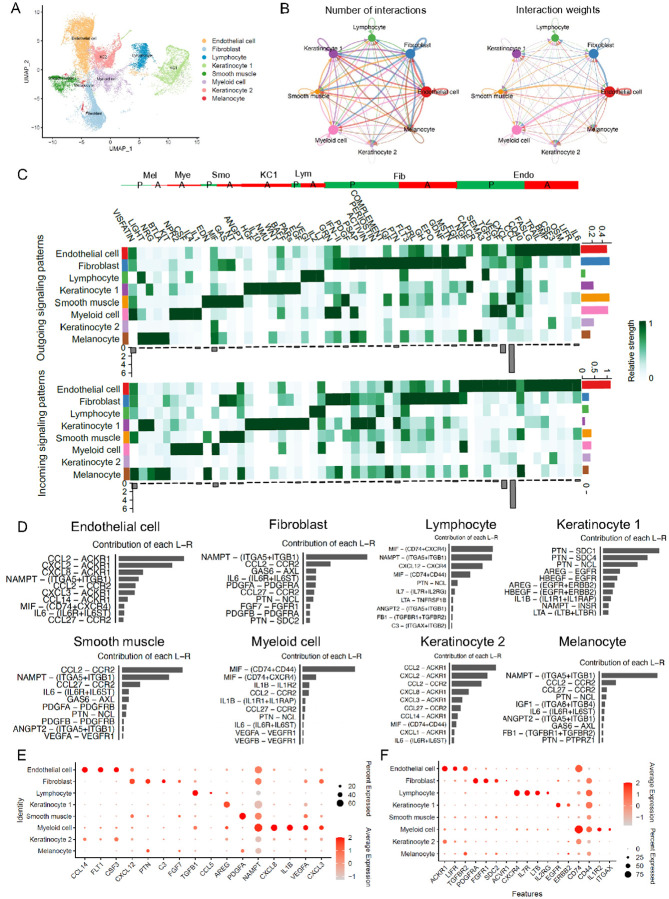
Overall Distribution of signaling pathway across cell clusters in normal skin. **A**) UMAP visualization identifies eight cell subpopulations from six donors. **B**) Circle plots show the number of interactions (left) and the weights of these interactions weights (right) in all cell clusters. **C**) Heatmap illustrates the relative contribution of each cell group to outgoing or incoming signals. P: Paracrine manner; A: Autocrine manner. Endo: Endothelial cell, Fib: Fibroblast, Lym: Lymphocyte, KC1: Keratinocyte 1, Smo: Smooth muscle cell, Mye: Myeloid cell, Mel: Melanocyte. **D**) The top 10 L-R pairs from each cell type based on their relative contribution under incoming patterns. **E-F**) Dot plot showing three datasets shared specific ligands (E) and receptors (F) genes for each cell type. The color scale represents the scaled expression average of each gene, while the dot size represents the percentage of cells expressing each gene.

To investigate the cell-cell communication in acne-affected skin, we first sought to display the overall signal distributions in normal skin samples as a reference point. Although several studies have explored the cell-cell interactions in aging, wound healing, psoriasis, and dermatitis in both mice and humans, there remains a gap in understanding the overall signal distribution in distinct cell types in normal human skin, which can be used as a baseline to compare with various skin diseases^2, 3^. To bridge this gap, we leveraged our previously published dataset, which sampled normal skin from the back of six individuals with active early-stage acne vulgaris, approximately 24 hours of onset. The dataset consists of 29,202 cells of 8 different types: endothelial cells (ECs), fibroblasts, lymphoid cells, smooth muscle, myeloid cells, two populations of keratinocytes (KCs) (Keratinocyte 1 and Keratinocyte 2), and melanocytes (Fig. 1A **and Figure S1A**). In our analysis, we identified KC1s as typical epithelial cells based on their specific expression of KRT14, KRT10, and KRT5. Conversely, KC2 was classified as sweat gland cells due to their high expression of cell markers such as KRT18, KRT19, KRT7, AQP5, and CEACAM5 (**Figure S1B**)^10, 11^. Using CellChat, we further analyzed this dataset to infer the cell-cell communication network, which revealed that all cell groups actively engage in mutual signaling with each other. Notably, ECs, fibroblasts, and myeloid cells showed the most significant number of interactions and the largest weights of cell interactions, which likely correlates with the high abundance of these cell types in the skin (Fig. 1B and S1C).

To understand the signal pathway distributions in each cell cluster and where these signals target, we analyzed the key incoming and outgoing signal patterns, detecting a total of 55 distinct signals. Outgoing patterns illustrate the distribution of signals secreted by sender cells and how the weights are distributed, whereas the incoming patterns reflect the reception of signals sent from others (Fig. 1C). Within these signaling pathways, some featured the expression of both ligands and receptors within the same cell type. We categorized these autocrine pathways as follows: I) IL-6, LIFR, OSM, CSF3, BMP, TRAIL, and FASLG; II) FGF, MSTN, GDNF, EPO, GH, PRL, and FLT3; III) LT and IL-2; IV) EGF, PARs, NMU, IL-10, HGF, BAFF, and WNT; V) NT and ANGPT; VI) IL-1, IL-4, CSF, and NPR2; VII) KIT, and BTLA. Each pathway operates within endothelial cells, fibroblasts, lymphocytes, keratinocytes, smooth muscle cells, myeloid cells, and melanocytes, respectively, serving diverse functions and establishing signaling circuits that support processes such as tissue development, cell survival, regulation of inflammation, immune response, and cell death^12–15^.

Simultaneously, every cell type communicates with others in a paracrine manner. ECs have long been known to maintain vascular homeostasis and provide paracrine support to surrounding non-vascular cells, as well as modulating inflammation by regulating immune cell trafficking, activation status, and function^16, 17^. Compared to other cell types, we particularly observed that ECs sent the strongest CD40 signaling out while receiving signals such as CCL, CXCL, VEGF, TGF-β, SEMA3, CALCR, and NGF signals from other cell types (Fig. 1C). Fibroblasts were identified as the strongest source of signals such as PTN, IGF, COMPLEMENT, PERIOSTIN, ACTIVIN, PSAP, and NGF to other cell types, and they predominantly received PDGF, IFN-II and GRN signals, highlighting their role in tissue homeostasis and disease through growth factors/hormone production and extracellular matrix formation^18, 19^. Smooth muscle cells were notable for sending signals like CCL, MIF, PDGF, GAS and EDN, which are crucial for involuntary muscle contractions and in regulating physiological processes such as blood flow^20^. As predominant immune cells in the skin, lymphocytes and myeloid cells were identified as strong sources of TGFβ VEGI, CXCL, VISFATIN, VEGF, IFN-II, and GRN signals, while primarily receiving MIF and COMPLEMENT signals. Keratinocytes are key component of the skin barrier and structural cells. They were the strongest source of CALCR signals, receiving various strong signals including PTN, PERIOSTIN, NRG, ACTIVIN, LIGHT, PSAP, and VEGI. Lastly, melanocytes, which are responsible for skin and hair pigmentation, sent out the strongest SEMA3, NRG and LIGHT signals, and received the strongest VISFATIN, IGF, EDN and CD40 signals, underlining their critical role in determining skin color and hair characteristics.

As every signal pathway consists of numerous ligand-receptor pairs, we further narrowed down our scope and focused on identifying key ligand and receptor genes that were not only from the strongest pathways but also exhibited unique expression within specific cell clusters. To achieve this, we analyzed all ligand-receptor pairs and their relative contribution within each cluster (**Figure S2-S9**). Through this comprehensive approach, we identified specific ligand and receptor genes by compiling them from the top three outgoing and incoming signals (Table 1), as well as the top ten ligand-receptor pairs contributed by each cluster (Figs. 1D and S10A). Subsequent validation of these gene expression patterns against two additional skin datasets confirmed the consistency of our findings (Figs. 1F–1E, **Figures S10B-S10C, and Figures S11-S17**)^21, 22^. In endothelial cells, we identified ligands (FLT1^23^, CCL14^24^, CSF3^25^) and receptors (ACKR1^26^, LIFR^14^, TGFBR2^27^), that are either known cell markers for endothelial cells or are involved in processes such as angiogenesis, migration, immune cell recruitment, vascular integrity (**Figures S11**). We found ligands (CXCL12^28^, PTN^29^, C3^30^, FGF7^31^) and receptors (PDGFRA^32^, SDC2^33^, ACVR1^34^, FGFR1^35^) were mainly expressed in fibroblasts (**Figures S12-S13**). Ligands TGFB1, CCL5 and receptors CXCR4, IL-7R, LTB, IL-2RG, and ITGB2 were found to be highly expressed in lymphocytes (**Figures S14**). In myeloid cells, enriched ligands (NAMPT, CXCL8, IL1B, VEGFA, and CXCL3) and the receptors (CD74, CD44, IL-1R2 and ITGAX) were identified (**Figures S15-S16**). KC1 showed enrichment for ligand AREG and receptors (EGFR and ERBB2) (**Figures S17A-S17B**). Smooth muscle cells showed distinct expression of ligand PDGFA (**Figures S17C**). No signaling pathway-associated genes specific to KC2 and melanocytes were identified. These findings suggest that genes derived from signaling pathways could serve as additional markers for cell cluster annotation in the skin.

#### Acne triggers significant signaling pathway changes across all cell clusters within the skin.

**Figure 2:**
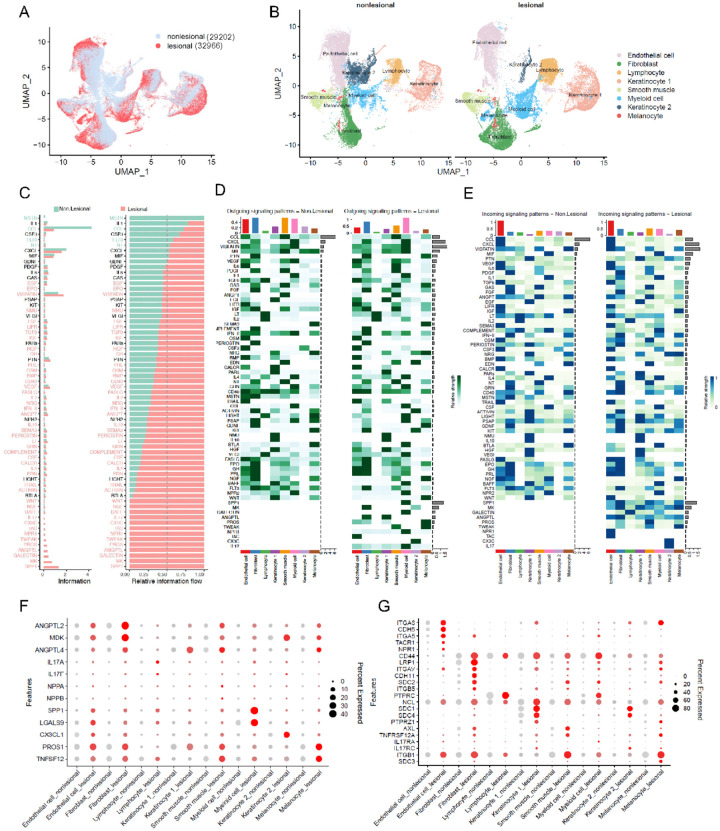
Acne triggers signaling pathway changes within the skin across all cell clusters. **A**) UMAP plot for nonlesional and lesional cells colored by lesional types, nonlesional cells are shown in light blue, and lesional cells are shown in red. **B**) UMAP plot showing the nonlesional and lesional samples split by lesional types. **C**) All detected signaling pathways were ranked based on the differences of overall information flow within the inferred networks between nonlesional and lesional samples. The signaling pathways in green show higher enrichment in nonlesional samples, those in black have similar enrichment in both conditions, and those in red are more prevalent in lesional samples. Bar graphs are displayed in both non-stacked (left) and stacked (right) formats. **D-E**) Heatmap show the comparison of outgoing (D) or incoming (E) signaling associated with each cell type between nonlesional and lesional samples. **F-G**) Dot plot showing the representative ligands (F) or receptors (G) genes from ten signaling pathway which were turned on in lesional compared with nonlesional samples. The color scale represents the scaled expression average of each gene, and the dot size represents the percentage of cells expressing each gene.

To elucidate the changes of cell-cell interactions from nonlesional to lesional samples, we initially integrated the datasets of nonlesional and lesional samples (Fig. 2A), and as demonstrated by Tran *et al*. we also observed significant changes in cellular compositions, especially in KC2 and fibroblasts^6^ (Fig. 2B). Further analysis of each signaling pathways revealed an increase in both the strength and number of signaling pathways in lesional compared to nonlesional samples (**Figure S18A-S18B**). We identified changes in 49 signal distributions: (i) one signal was turned off (MSTN), (ii) three signals were decreased (CCL, FLT3, NT), (iii) ten signals were turned on (IL-17, CX3C, TAC, NPR1, TWEAK, PROS, ANGPTL, GALECTIN, MK and SPP1), and (iv) thirty-five signals were increased (including BAFF, NGF, WNT) (Fig. 2C). We found that these signal changes involved all cell clusters, indicating the possibility that immune responses within acne skin trigger responses across every cell type (Fig. 2D–2E). Of the ten signaling pathways that were turned on, IL-17, NPR1, GALECTIN and SPP1 mainly derived from myeloid cells and targeted KC2, endothelial cells, lymphocytes, and fibroblasts, respectively. The presence of IL-17 signaling in acne, is consistent with our previous findings^36^. PROS and TWEAK signals originated from melanocytes and can target both smooth muscle and melanocytes. A case-controlled study of 100 acne vulgaris patients reported that acne patients had significant elevation in TWEAK serum levels when compared to the control subjects, which is consistent with our findings^37^. TAC and CX3C signals interact in an autocrine way in endothelial cells and KC2. MK signals mainly from fibroblasts target melanocytes, whereas ANGPTL signals from KC1 target fibroblasts. These changes occurred across all cell types were further supported by the observed significant increase in the expression level of ligands and receptors associated with the ten turn-on signaling pathways in lesional samples of acne (Fig. 2F–2G).

#### Activation of GRN and IL-13RA1-related signals.

**Figure 3:**
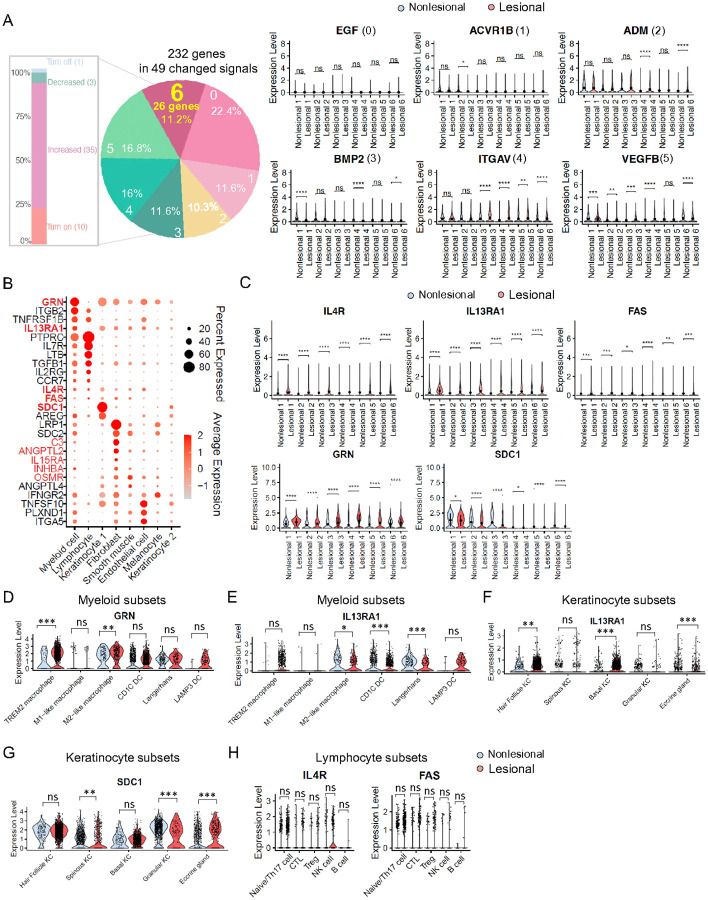
GRN and IL-13RA1-related signals are activated among individuals. **A**) Histogram showing the percentage and number of altered signaling pathways in lesional compared with nonlesional samples (left). Pie chart showing the seven distribution possibilities and percentage of 232 genes in six donors, with numbers 0 to 6 and corresponding percentiles indicating certain percentage of genes that are significantly changed in 0 to 6 donors. Violin plots illustrate the expression changes of representative genes (*EGF, ACVR1B, ADM, BMP2, ITGAV, VEGFB*), which are significantly altered in 0 to 5 donors in lesional compared to nonlesional samples. Wilcoxon test was used to perform the statistical analysis (right), ns (not significant), *P < 0.05, **P < 0.01, ***P < 0.001, ****P < 0.001. **B**) Dot plot shows 26 genes significantly altered across six donors; 10 genes highlighted in red indicate consistent expression trends across all matched pairs in all patients. Among these, 5 are bolded to indicate their specific expression in keratinocytes, lymphocytes, and myeloid cells. The color scale indicates the average scaled expression of each gene, and the dot size reflects the percentage of cells expressing each gene. **C)** Violin plots reveal five genes (*IL-4R, IL-13RA1, FAS, GRN, SDC1*) consistently and significantly changed among the six donors, analyzed using the Wilcoxon test, *P < 0.05, **P < 0.01, ***P < 0.001, ****P < 0.001. **D-E)** Violin plot showing the *GRN* (D) and *IL-13RA1*(E) expression in subsets of myeloid cells. Wilcoxon test was used to perform the statistical method, ns (not significant), *P < 0.05, **P < 0.01, ***P < 0.001. **F-G)** Violin plot showing the *IL-13RA1*(F) and *SDC1* (G) expression in subsets of keratinocytes. Wilcoxon test was used to perform the statistical method, ns (not significant), **P < 0.01, ***P < 0.001. **H)** Violin plots showing the *IL-4R* and *FAS* expression in subsets of lymphocytes. Wilcoxon test was used to perform the statistical method, ns (not significant).

To account for the diversity in signaling pathway alterations observed across different donors and the impact of outliers, we aimed to identify significant differences in gene expression within each matched pair of nonlesional and lesional samples from six donors. We performed a differential analysis on all 232 genes associated with the 49 altered signaling pathways, these genes exhibited seven distinct expression profiles (Fig. 3A). Among them, 26 genes (11.2%) displayed significant differences between nonlesional and lesional samples within each patient (Fig. 3B and **Figure S19**). Conversely, 52 genes (22.4%) showed no differences across all six individuals, while 27 (11.6%), 24 (10.3%), 27 (11.6%), 37 (16%), and 39 (16.8%) genes exhibited significant differences in 1, 2, 3, 4, and 5 matched nonlesional and lesional sample pairs, respectively (Fig. 3A). Previous studies have indicated that papules can form in under 6 hours and exhibit a profound inflammatory response as evidenced by increased levels of CD4 T cells, neutrophils, and CD68^+^ macrophages in acne biopsies. However, KC did not exhibit abnormal proliferation compared to normal skin at that time point^38^. Given that our samples were collected from patients at approximately 24 hours into the disease course, later than the 6 hour-mark, we focused on genes linked to signaling pathways in lymphocytes, myeloid cells, and basal cells in KC. These genes may be associated with inflammation and hyperkeratinization during this period. In these three cell types, only 5 genes (*GRN, IL13RA1, IL4R, FAS* and *SDC1*) showed consistent expression patterns across all matched pairs in all patients (Fig. 3C). Among these, GRN, also known as the granulin precursor and a multifunctional growth factor, has been identified in macrophages across various organs, including the lung and brain^39, 40^. GRN plays a dual function in regulating inflammation and is associated with processes such as tumorigenesis, neurodegeneration, wound healing, and early embryogenesis. In our dataset, *GRN* primarily originates from myeloid cells including TREM2 macrophages, M1 and M2 macrophages, CD1C dendritic cells (DCs), Langerhans and LAMP3 DCs (**Figure S20A-S20B**). Notably, we observed higher expression of *GRN* in TREM2 macrophages and M2-like macrophages in lesional skin compared to nonlesional skin (Fig. 3D). Given that TREM2 macrophages have been implicated in driving inflammation in acne^6^, our subsequent analyses focused on the function of GRN within these cells.

*IL-13RA1* was also upregulated in lesional skin, primarily originating from myeloid cells (Fig. 3C). However, it was either downregulated or showed no significant difference in subsets of myeloid cells, implying that increased expression levels of *IL-13RA1* came from other cell types in lesional skin (Fig. 3E). Therefore, our focus shifted to the second largest source of *IL-13RA1*, which was KC1. Specifically, we focused on basal cells from KC1 given their critical role in skin self-renewal and their significant involvement in hyperkeratinization within the epidermis. We found that *IL-13RA1* was markedly upregulated in lesional basal cells compared to nonlesional ones (Fig. 3F, **Figure S20C-S20D**). This observation aligns with a study suggesting that IL-13, produced by group 2 innate lymphoid cells in the crypt niche, interacts with IL-13RA1 on Lgr5^+^ intestinal stem cells^41^, suggesting potential involvement of IL-13RA1 in hyperkeratinization during acne development. Additionally, *SDC1* was highly expressed in KC1, but showed no significant difference in basal cells between the two conditions (Fig. 3G). *IL-4R* and *FAS* were predominantly expressed in lymphocytes; however further analysis revealed that neither *IL-4R* nor *FAS* showed significant changes in lymphocytes subsets (Fig. 3H **and Figure S20E-S20F**). Consequently, we chose not to investigate these three genes further.

**Figure 4:**
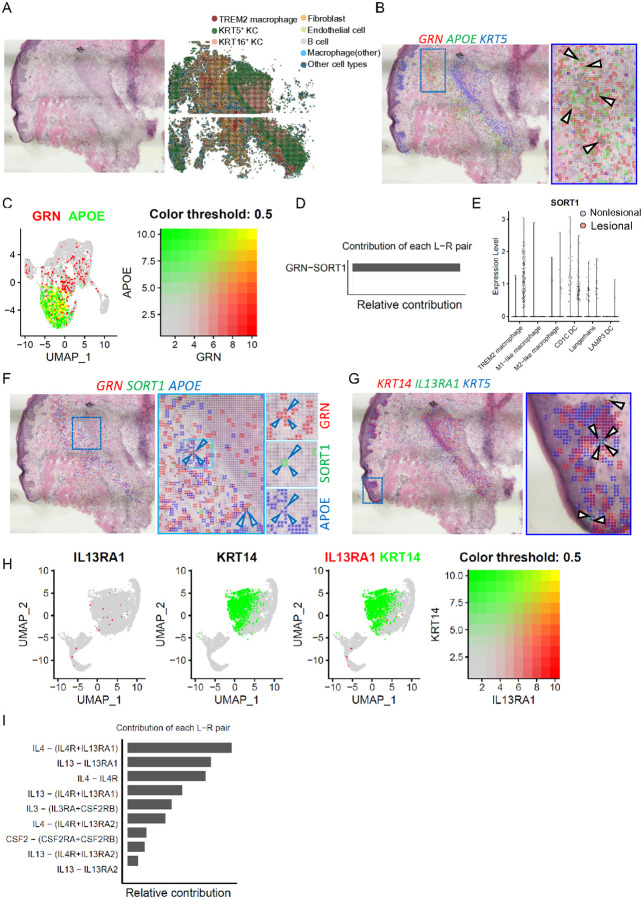
Spatial transcriptome sequencing reveals the localization of *GRN* and *IL-13RA1* in skin biopsy. **A)** H&E staining image of the acne biopsy used for Seq-Scope sequencing (left) alongside a spatial plot that identifies eight cell clusters in acne lesion (right). **B)** Spatial feature plot showing overlay of *GRN* in red, *APOE* in green, and *KRT5* in blue with 2-μm intervals between grids; boxed region showing the magnified spatial plot. **C)** UMAP plot showing the co-expression of *GRN* in red and *APOE* in green in scRNA-seq dataset. **D)** Analysis of the relative contribution of the *GRN-SORT1* ligand-receptor (L-R) pair within the GRN signaling communication network. **E)**Violin plot showing the *SORT1* expression in subsets of myeloid cells. **F)** Spatial feature plot showing the overlay of *GRN* in red, *SORT1* in green, and APOEin blue with 2-μm intervals between grids; boxed region shows the magnified spatial plot. **G)** Spatial feature plot showing the overlay of *IL-13RA1* in green, *KRT14* in red, and *KRT5* in blue with 2-μm intervals between grids; boxed region showing the magnified spatial plot. **H)** UMAP plot showing the co-expression of *IL-13RA1* in red and *KRT14* in green in the scRNA-seq dataset. **I)** Relative contribution of each L-R pair to the overall communication network of IL-4 signaling pathway.

Next, to spatially localize GRN and IL-13RA1 expression in acne skin, we used the Seq-Scope sequencing dataset obtained from acne lesions and segmented the histological area using 10 μm-sided square grids^6^. The analyzed specimen featured a hair follicle surrounded by an inflammatory infiltrate. Each grid detected an average of 145 genes across 3558 grids, enabling the identification of eight distinct cell populations including KRT5 and KRT16 keratinocytes, fibroblasts, endothelial cells, TREM2 macrophages, B cells, other macrophages, and various other cell types. (Fig. 4A). Our initial focus on *GRN* expression revealed its prominence in TREM2 macrophage where it co-localized with TREM2 macrophage marker, *APOE*, both in scRNA-seq and Seq-Scope dataset (Fig. 4B–4C). To identify the receptor for GRN, we analyzed the contribution of all ligand-receptor (L-R) pairs in GRN signaling. Our findings revealed that only one receptor, Sortilin (*SORT1*), was detected and significantly upregulated in TREM2 macrophages. Furthermore, SORT1 was found to colocalize with *GRN* in acne lesions (Fig. 4D–4F). This L-R binding was first identified in the brain, underscores SORT1’s role in mediating rapid endocytosis and lysosomal localization of GRN, central in the development of inherited frontotemporal lobar degeneration^42^. Other studies have also shown that both GRN and SORT1 are key regulators of inflammation^43, 44^. Our findings showing *GRN*^+^ cells also expressing *SORT1* in lesions, suggest that the GRN-SORT1 axis functions in an autocrine manner within TREM2 macrophages. Additionally, *IL-13RA1* was co-localized with basal KCs, marked by *KRT14* and *KRT5*^45,46^, consistent with the scRNA-seq data (Fig. 4G–4H). Subsequent analysis of the relative contribution of each L-R pair revealed that both IL-4 and IL-13 ligands can interact with IL-13RA1 (Fig. 4I). Subsequently, IL-13RA1 may be regulated by IL-4 and IL-13 in the basal KCs of the skin.

#### Activation of GRN and IL-13RA1 exacerbates inflammation and hyperkeratinization both of which are critical in acne progression.

**Figure 5:**
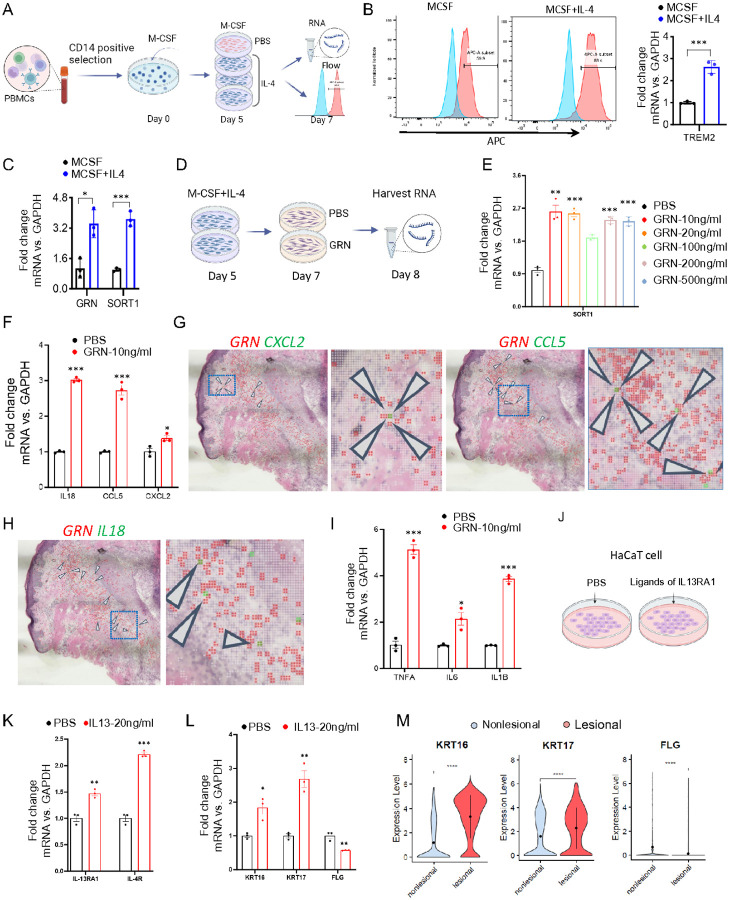
Activation of GRN and IL-13RA1 exacerbates inflammation and hyperkeratinization. **A)** Schematic diagram showing the process of generating TREM2 macrophages from human blood and detection by qRT-PCR and Flow cytometry. **B)** Flow cytometry (left) and qRT-PCR (right) analysis for the expression level of *TREM2*, ***P < 0.001 (Student’s t-test), n = 3 donors. **C)** qRT-PCR analysis for the expression level of *GRN* and *SORT1* in CD14 positive cells treated with or without recombinant IL-4 protein from day 5 to day 7, *P < 0.05, ***P < 0.001 (Student’s t-test), n = 3 donors. **D)** Schematic diagram showing the treatment of TREM2 macrophages with GRN and PBS. **E)** qRT-PCR analysis for the expression level of *SORT1* in TREM2 macrophages treated with different concentration of GRN and PBS for 24 hrs, **P < 0.01, ***P < 0.001 (Student’s t-test), n = 3 donors. **F and I**) qRT-PCR analysis for the expression level of proinflammatory cytokines *IL-18, CCL5, CXCL2* (F) and *TNF-α, IL-6, IL-1β* (I) in TREM2 macrophages treated with 10 ng/ml GRN and PBS for 24 hrs, *P < 0.05, **P < 0.01, ***P < 0.001 (Student’s t-test), n = 3 donors. **G-H)** Spatial feature plot showing the overlay of *CXCL2, CCL5, IL-18* in green, and *GRN* in red with 2-μm intervals between grids in acne lesion, with arrows indicating the colocalization of double positive spots, boxed region showing the magnified spatial plot. **J)** Schematic diagram showing the treatment of PBS, IL-4, and IL-13 in HaCaT cell line. **K-L)** qRT-PCR analysis for the expression level of *IL4R IL13RA1* (K) and *KRT16, KRT17, FLG* (L) in HaCaT cell line treated with PBS and IL-13, *P < 0.05, **P < 0.01, ***P < 0.001 (Student’s t-test), n=3 technical replicates. **M)** Violin plots showing *KRT16, KRT17*, and *FLG* expression in HaCaT cell line treated with PBS and IL-13. Wilcoxon test was used to perform the statistical method, ***P < 0.001, ****P < 0.0001.

Next, to explore the function of GRN in TREM2 macrophages, we induced TREM2 macrophage differentiation *in vitro* using macrophage colony-stimulating factor (M-CSF) and IL-4 as previously described (Fig. 5A)^6, 47^. We observed that the combination of M-CSF/IL-4 induced higher TREM2 expression compared to M-CSF alone (Fig. 5B). Further analysis revealed that both *GRN* and *SORT1* were upregulated in MCSF/IL-4-induced TREM2 macrophages, suggesting that GRN may play a significant role in TREM2 macrophages activation through its interaction with SORT1 (Fig. 5C). Tran *et al*. reported that TREM2 macrophages elicit a proinflammatory response by increasing the expression of proinflammatory cytokines and chemokines, such as IL-18, CCL5, and CXCL2^6^. To investigate the involvement of GRN in the proinflammatory activity of TREM2 macrophages, we treated these cells with recombinant GRN protein, which induced SORT1 expression (Fig. 5D). Our results demonstrated that treatment with 10 ng/ml of GRN activated the upregulation of *SORT1*. Intriguingly, higher concentrations of GRN did not enhance the *SORT1* response, prompting the selection of 10 ng/ml of GRN as the optimal concentration for further studies (Fig. 5E). This treatment also elevated levels of proinflammatory cytokines (IL-18, CCL5, and CXCL2) known to activate the canonical inflammatory NF-kB pathway, recruiting T cells, mast cells, and natural killer cells, as well as promoting neutrophil infiltration^48–50^ (Fig. 5F). These observations were corroborated by the colocalization of *GRN*^+^ cells with IL-18, CCL5, and CXCL2-expressing cells in acne lesions (Fig. 5G–5H). Additionally, we observed that GRN promotes the expression of proinflammatory cytokines (*TNFA, IL-TB*, and *IL-6*) in TREM2 macrophages (Fig. 5I), and the coexpression of *TNFA* and *IL-1B* can be found within *GRN*^+^ cells (**Figure S21A-S21B**). Altogether, these data suggest that GRN amplifies the inflammatory response in TREM2 macrophages.

Hyperkeratinization, a key initial event in microcomedone formation, can be caused by anomalies in the differentiation, adhesion, and proliferation within the follicular infundibulum. Molecular markers such as KRT6, KRT16 and KRT17 are upregulated, whereas filaggrin (FLG), a marker for keratinocyte differentiation, is downregulated in established microcomedones^51–53^. To investigate the role of IL-13RA1 in hyperkeratinization, we activated IL-13RA1 in the keratinocyte cell line (HaCaT) with its ligands IL-13 and IL-4. Our findings revealed that compared to the control group, IL-13 treatment significantly upregulated the expression of both *IL-13RA1* and *IL-4R* in HaCaT cells (Fig. 5J–5K). In contrast, IL-4 treatment either did not alter or downregulate *IL-13RA1* and *IL-4R* expression (**Figure S21C**), indicating that only IL-13 activate *IL-13RA1* in keratinocytes, which is consistent with the findings in intestinal epithelial cells and bone marrow-derived macrophage^41,54^. Subsequent analysis showed that IL-13 treatment led to increased expression of *KRT16* and *KRT17*, accompanied by reduced *FLG* expression, these gene expression patterns were consistent with our scRNA-seq data (Fig. 5L–5M). However, no significant change was observed in *KRT6A* expression (**Figure S21D**). Collectively, these data suggest that IL-13RA1 signaling may play a significant role in driving the dysregulation of genes related to hyperkeratinization, contributing to the development of acne in human skin.

## Discussion

**Figure 6:**
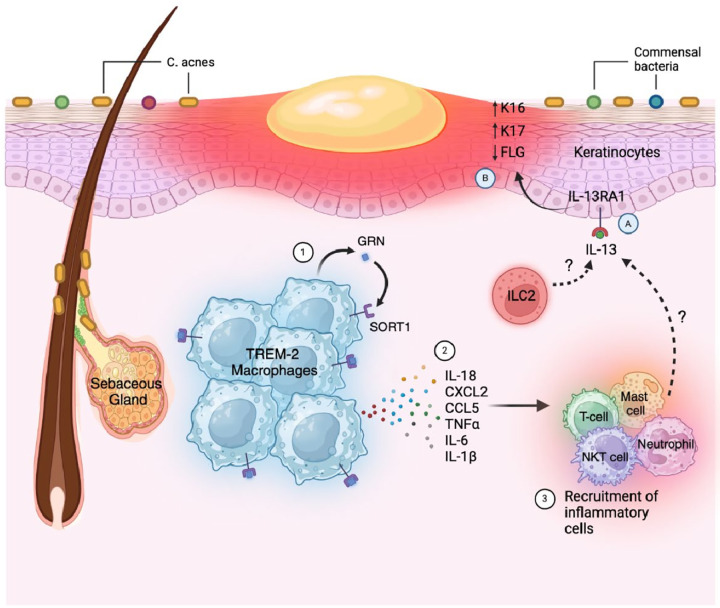
A schematic overview highlighting the critical role of GRN in promoting inflammatory responses in TREM2 macrophages and IL-13RA1 in modulating the expression of genes associated with hyperkeratinization in acne skin. In acne lesions, resident TREM-2 macrophages release granulin precursor (GRN) (1), which binds to their surface receptor SORT1 and (2) stimulates the release of proinflammatory cytokines such as IL-18, CXCL2, CCL5, TNF-α, IL-6, and IL-1β. (3) These cytokines recruit inflammatory cells such as mast cells, T cells, NKT cells, and neutrophils. Besides its role in inflammation, this figure also depicts GRN’s possible role in hyperkeratinization. (A) Recruited inflammatory cells together with ILC2s, secrete IL-13, which binds to the IL-13RA1 receptor on keratinocytes (B), leading to the upregulation of K16 and K17–markers associated with keratinocyte hyperproliferation–as well as the downregulation of FLG.

In our study, we first investigated the distribution of signaling pathways within different cell types in both normal and acne skin. Through detailed analysis, we identified 49 signaling pathways that were altered in acne, along with genes showing consistent expression changes across all donors. Our subsequent focus centered on examining the roles of GRN in TREM2 macrophages and IL-13RA1 in keratinocyte basal cells given their consistent alterations across donors and potential importance in acne development. Using spatial-seq datasets, we confirmed the expression and colocalization of these genes with their respective cell types in acne samples. Further exploration of their functional roles *in vitro* revealed that GRN may exacerbate acne progression by enhancing inflammation in TREM2 macrophages, as demonstrated by its induction of inflammatory cytokines and chemokine expression. Conversely, the upregulation of IL-13RA1 in basal cells suggests its potential involvement in hyperkeratinization. We activated IL-13RA1 by IL-13 in the HaCaT cell line, which resulted in the dysregulation of genes associated with hyperkeratinization, further implicating its role in acne development. Together, our findings shed light on the complex interplay between inflammation and hyperkeratinization in acne pathogenesis, while also highlighting GRN and IL-13RA1 as promising therapeutic targets for acne (Fig. 6).

The initial phase of acne is characterized by the presence of microcomedones, which progresses into papules, pustules, nodules, and cysts as the severity worsens. Studies have indicated the involvement of various innate and adaptive immune cells, including Th1^55^, Th17^56^, Foxp3^+^, CD1^+^, CD83^+^ DCs^57^, CD68^+^ macrophages, and activated mast cells in the early events, along with the secretion of proinflammatory cytokines and chemokines. Limited studies have comprehensively explored dysregulated signaling pathways in different skin cell clusters. In our study, we detected 49 altered signaling pathways encompassing 232 genes in lesional samples compared to nonlesional samples across all cell clusters. Subsequent analysis revealed that not all these genes exhibit significant changes in all donors. However, we identified 10 genes that were consistently dysregulated in all donors and specifically expressed in lymphocytes, myeloid cells, keratinocytes, fibroblasts, and smooth muscle. Among these, Dahl *et al*. observed C3 presence at the dermo-epidermal junction in the majority of inflammatory acne lesions, contrasting with non-inflammatory samples^58^. This observation was further supported by Scott *et al.*, who associated early complement activation with acne inflammation^59^, and these findings also align with our results that fibroblast-derived C3 is upregulated in acne. The A > G polymorphism in the IL-4R gene has been associated with heightened allergic and immune-mediated disorders^60^. In a study by Robaee *et al.*, a comparison of genetic polymorphisms in IL-4R between 95 acne patients and 87 unrelated healthy controls revealed a significant difference in IL-4R (Q551R A/G) genotypes between the two groups^61^, yet its role in acne remains unknown. In our data, *IL-4R* was mainly expressed in lymphocytes, but no significant difference was found in lymphocyte subsets, so further investigation is needed to understand its function in other cell types. Moreover, the function of the other genes, including FAS, SDC1, ANGPTL2, IL-15RA, INHBA, and OSMR in lymphocytes, keratinocytes, fibroblasts, and smooth muscle, are yet to be explored, suggesting that acne involves not only the skin’s surface but also a wider systemic dysregulation. Future studies should focus on these genes to advance our understanding of acne pathogenesis.

Recent research has extensively investigated the specific expression of GRN and TREM2 on microglia, the brain-resident macrophages, revealing their links to neurodegenerative disorders such as frontotemporal lobar degeneration and Alzheimer’s disease^62, 63^. However, Götzl *et al*. discovered that microglia isolated from GRN^−/−^ mice exhibited a hyperactivated state of the neurodegenerative phenotype molecular signature and suppression of genes characteristic of homeostatic microglia. Conversely, loss of TREM2 enhanced the expression of genes associated with a homeostatic state but reduced glucose metabolism in both conditions. This suggests that opposite microglial phenotypes lead to similar widespread brain dysfunction^64^. Our *in vivo* data initially revealed GRN expression predominantly in myeloid cells, with a significantly higher expression in TREM2 macrophages in lesional compared to nonlesional samples. Additionally, colocalization of *GRN and* TREM2 macrophages was observed in spatial-seq data. Further investigation detected higher expression of *GRN* in IL-4-induced TREM2 macrophages compared to non-IL-4-treated cells, indicating a strong correlation between GRN and TREM2 macrophages.

The role of GRN in inflammation is diverse, showing variability across different disease conditions, tissues, and even cell types. A wealth of evidence from *in vitro* and animal models suggests that GRN possesses anti-inflammatory properties. GRN competitively binds with TNFR1/2 to disrupt TNF-α function, which in turn leads to increased IL-10 production in T regulatory cells in conditions such as rheumatoid arthritis and inflammatory bowel disease^65, 66^. Additionally, GRN can selectively inhibit the release of TNF-α and IFN-γ-induced CXCL9 and CXCL10 in CD4^+^ T cells^67, 68^. However, the interaction between GRN and TNFR1/2 appears to be complex, with some studies suggesting that GRN does not bind to TNF receptors, thus not directly influencing TNF signaling in various cell lines^69–71^. On the contrary, GRN can exhibit a pro-inflammatory effect by promoting the expression of proinflammatory cytokines such as IL-6 and IL-8 in different diseases such as psoriasis, obesity, and systemic lupus erythematosus^72–76^. These contradictory findings suggest that GRN possesses characteristics of a double-edged sword in inflammation, acting both as a protector and provocateur depending on the condition. Our studies on the effect of recombinant GRN on TREM2 macrophages indicate that GRN activates its receptor SORT1 and promotes the expression of proinflammatory cytokines and chemokines from TREM2 macrophages, thereby activating downstream NF-kB signaling pathways^48–50^. These findings suggest that the proinflammatory function of TREM2 macrophages can be driven through the GRN-SORT1 axis.

IL-13RA1 serves as the receptor or coreceptor for IL-13 and IL-4, playing a critical role in type 2 immunity, which encompasses both host-protective and pathogenic functions^77^. Our data revealed that IL-13RA1 levels were either downregulated or remained unchanged in certain myeloid subsets, a trend contrary to that observed in whole-sample analyses. Therefore, we redirected our focus towards its role in keratinocytes and observed that IL-13RA1 was notably upregulated in basal cells. Previously, studies have demonstrated that IL-13 promotes the self-renewal of intestinal stem cells solely through IL-13RA1 but not IL-4R, underscoring the proliferative function of the IL13-IL13RA1 axis^41^. In the skin, IL-13 activation of IL-13RA1 disrupts the skin’s barrier function and facilitates terminal differentiation by downregulating the expression levels of epidermal barrier proteins such as FLG, loricrin (LOR), and involucrin in primary human epidermal keratinocytes^78–80^. Our findings align with these observations, as we discovered that IL-13 activation of IL-13RA1 resulted in the downregulation of *FLG* expression and upregulation of hyperproliferation-associated keratins KRT16 and KRT17^81, 82^. Thus, our data suggests that the IL-13-IL-13RA1 axis significantly influences keratinocyte proliferation and plays a key role in acne pathogenesis. Interestingly, TREM2 macrophages-recruited mast cell, NKT cell, T cell and neutrophils, all capable of secreting IL-13^83, 84^, this connection bridges inflammation and hyperkeratinization processes in acne, indicating that inflammation precedes and triggers hyperkeratinization. Our data collectively suggest that inflammation and hyperkeratinization, driven by common dysregulated GRN and IL13RA1 may be pivotal in acne development. Targeting these pathways holds promise for more effective acne treatments.

## Materials and Methods

### PBMC and monocyte isolation

PBMCs were obtained from the blood of healthy donors after signed written informed consent as approved by the Institutional Review Board at UCLA following the Helsinki Guidelines. PBMCs were then isolated using Ficoll–Paque density gradients (GE Healthcare) as previously described^36^. Monocytes were isolated from PBMC by positive selection with CD14 MicroBead (Miltenyi Biotec, Cat#130-050-201), then seeded at 800,000 cells per well in 12-well plates.

### TREM2 macrophage differentiation and evaluation via flow cytometry

CD14 positive cells were differentiated in M-CSF (50 ng/ml) (R&D Systems, Cat#216MC025/CF) for 5 days in RPMI 1640 with 10% FBS at 37°C. To differentiate to TREM2 macrophage, IL-4 (100 ng/ml) (R&D Systems, Cat#204-IL-020/CF) was added from day 5. On day 7, TREM2 expression was evaluated via flow cytometry. Briefly, adherent cells were detached with 1 mM EDTA in PBS and stained with mAbs against TREM2 (R&D Systems, Cat# FAB17291A). Isotype control staining was performed in parallel. Cells were acquired with an LSR II flow cytometer (BD) and analyzed with FlowJo (BD).

### RNA isolation, cDNA synthesis, and real-time PCR

Total RNA was isolated using Trizol reagent (Thermo Fisher, Cat#15596018) following manufacturer’s protocol. RNA samples were reverse transcribed to cDNA using Script Reverse Transcription Supermix (Bio-Rad, Cat#1708841). Reactions were performed at 25°C for 5 min, 46°C for 20 min and 95°C for 1 min. Real-time PCR was applied using SensiFAST SYBR & Fluorescein Kit (Thomas Scientific, Cat#C755H99). 40 cycles were carried out at 95°C for 5 min, then 95°C for 10 sec, 60°C for 12 sec, 72°C for 12 sec. GAPDH was used as a control. The gene expression level was quantified by the comparative method 2^−ΔΔCT^. The primers used for gene assessment are summarized in Supplementary Table 2.

### HaCaT cell culture and treatment

DMEM + GlutaMAX^™^-I (Gibco, Cat#10566–016) containing Penicillin/Streptomycin, 10% FBS was used to culture the HaCaT cell line. HaCaT cells were seeded and grown in 12-well plates to ~ 60% confluent then using various concentrations of IL-4 (R&D Systems, Cat#204-IL-020/CF) and IL-13 (Thermo Scientific, 200-12-2UG) were then added. Cells were harvested after 24 hours and used for further experiments.

### Data and code availability

For the scRNA-seq data, the sample processing and analysis for this dataset were described in a previous study^6^, and downstream analysis (Data visualization, clustering, cell type mapping, subsetting) was performed according to the Seurat tutorial series (https://satijalab.org/seurat/articles/visualization_vignette), and cell-cell interaction and comparison analysis according to the CellChat tutorial series (https://github.com/jinworks/CellChat). The sample processing and analysis for the Seq-Scope spatial dataset were conducted as previously described^6 85^. Briefly, this dataset included a 6 mm punch biopsy from a back acne papule. This sample was frozen in OCT medium and stored at −80°C until sectioning. For the Seq-Scope array, HISEQ2500 flow cells were used instead of the usual MISEQ flow cells. The distinctions between these two types of flow cells can be found in^6^. Published seq-scope datasets, step-by-step protocol, and data processing tools of Seq-ScopeMISEQ and Seq-ScopeHISEQ will be available at http://www.seq-scope.com and updated regularly.

### Statistical analysis

Statistical analyses were performed using GraphPad Prism version 9.0, with *P* values ≤ 0.05 were assigned as significant. For comparisons between two groups, an unpaired Student’s *t test* with two-tailed *p*-value analysis was performed, unless otherwise stated in the figure legend.

### Study approval

This study was conducted according to the principles expressed in the Declaration of Helsinki. The study was approved by the UCLA IRB (no. 22–000400).

## Figures and Tables

**Table 1:** Top 3 outgoing and incoming signals from each cell type in normal skin samples

Outgoing signal	Incoming signal				
Sender	L-R pair	Receiver	Receiver	L-R pair	Sender
Endothelial cell	CCL2 – ACKR1	Endothelial cell	Endothelial cell	CXCL8 – ACKR1	Myeloid cell
	CCL14 – ACKR1	Endothelial cell		CCL2 – ACKR1	Smooth muscle
	CXCL2 – ACKR1	Endothelial cell		CXCL2 – ACKR1 (F-E)	Fibroblast
Fibroblast	CXCL2 – ACKR1	Endothelial cell	Fibroblast	NAMPT – (ITGA5 + ITGB1)	Myeloid cell
	CXCL3 – ACKR1	Endothelial cell		NAMPT – (ITGA5 + ITGB1)	Endothelial cell
	CCL2 – ACKR1	Endothelial cell		NAMPT – (ITGA5 + ITGB1)	Smooth muscle
Lymphocyte	CXCL12 – CXCR4	Lymphoid	Lymphocyte	NAMPT – (ITGA5 + ITGB1)	Myeloid cell
	MIF – (CD74 + CD44)	Myeloid cell		CXCL12 – CXCR4	Lymphocyte
	IL7 – (IL7R + IL2RG)	Lymphoid		NAMPT – (ITGA5 + ITGB1)	Endothelial cell
KC1	CCL27 – CCR2	Smooth muscle	KC1	PTN – SDC1	Fibroblast
	CCL27 – CCR2	Endothelial cell		PTN – SDC4	Fibroblast
	MIF – (CD74 + CD44)	Myeloid cell		PTN – NCL	Fibroblast
Smooth muscle	CCL2 – ACKR1	Endothelial cell	Smooth muscle	CCL2 – CCR2	Smooth muscle
	CCL2 – CCR2	Smooth muscle		CCL27 – CCR2,	Keratinocyte 1
	CXCL2 – ACKR1	Endothelial cell		CCL2 – CCR2	Endothelial cell
Myeloid cell	CXCL8 – ACKR1	Endothelial cell	Myeloid cell	MIF – (CD74 + CD44)	Smooth muscle
	CXCL2 – ACKR1	Endothelial cell		MIF – (CD74 + CD44)	Melanocytes
	CXCL3 – ACKR1	Endothelial cell		MIF – (CD74 + CD44)	Keratinocyte 2
KC2	CCL2 – ACKR1	Endothelial cell	KC2	CXCL8 – ACKR1	Myeloid cell
	CXCL2 – ACKR1	Endothelial cell		CCL2 – ACKR1	Smooth muscle
	MIF – (CD74 + CD44)	Myeloid cell		CXCL2 – ACKR1	Fibroblast
Melanocyte	CCL2 – ACKR1	Endothelial cell	Melanocyte	NAMPT – (ITGA5 + ITGB1)	Myeloid cell
	MIF – (CD74 + CD44)	Myeloid cell		NAMPT – (ITGA5 + ITGB1)	Endothelial cell
	CCL2 – CCR2	Smooth muscle		NAMPT – (ITGA5 + ITGB1)	Smooth muscle

**Table 2:** q-PCR primers

Gene name	sequence (5′ -> 3′)
*GAPDH*-F	CTGGGCTACACTGAGCACC
*GAPDH*-R	AAGTGGTCGTTGAGGGCAATG
*TREM2*-F	GGTCAGCACGCACAACTT G
*TREM2*-R	CGCAGCGTAATGGTGAGAGT
*GRN*-F1	CCCTGGCAAAGAAGCTCCC
*GRN*-R1	AGCTCACAGCAGGTAGAACCA
*SORT1*-F	GGGGACACATGGAGCATGG
*SORT1*-R	GGAATAGACAATGCCTCGATCAT
*IL13RA1*-F	GTCCCAGTGTAGCACCAATGA
*IL13RA1*-R	GCTCAGGTTGTGCCAAATGC
*KRT16*-F	GACCGGCGGAGATGTGAAC
*KRT16*-R	CTGCTCGTACTGGTCACGC
*KRT17*-F	GCCGCATCCTCAACGAGAT
*KRT17*-R	CGCGGTTCAGTTCCTCTGTC
*IL4R*-F	ACACCAATGTCTCCGACACTC
*IL4R*-R	TGTTGACTGCATAGGTGAGATGA
*KRT6A*-F	CTGAATGGCGAAGGCGTT
*KRT6A*-R	CTGCCGACACCACTGGC
*Filaggrin*-F	GGCACTGAAAGGCAAAAAGG
*Filaggrin*-R	AGCTGCCATGTCTCCAAACTA
